# Development and validation of the Healthcare Worker Stress Scale-Vietnamese: a culturally grounded instrument to assess work-related stress

**DOI:** 10.1080/16549716.2025.2576369

**Published:** 2025-10-31

**Authors:** Hanh Thi Kieu Le, Thao Thi Phuong Nguyen, Seung Won Lee

**Affiliations:** a Department of Environmental Health, Faculty of Public Health, Thai Binh University of Medicine and Pharmacy, Hung Yen, Vietnam; b Department of Research and Innovation, Academy of Medical Sciences, Ho Chi Minh City, Vietnam; c Department of Precision Medicine, Sungkyunkwan University School of Medicine, Suwon, Republic of Korea; d Department of Artificial Intelligence, Sungkyunkwan University, Suwon, Republic of Korea; e Department of Metabiohealth, Sungkyunkwan University, Suwon, Republic of Korea; f Personalized Cancer Immunotherapy Research Center, Sungkyunkwan University School of Medicine, Suwon, Republic of Korea

**Keywords:** Occupational health, health personnel, occupational stress, psychometrics, validation study

## Abstract

**Background:**

Reliable measurement of occupational stress is essential for designing effective interventions for healthcare workers; however, Vietnam currently lacks culturally validated assessment tools.

**Objectives:**

To develop and validate the Healthcare Worker Stress Scale – Vietnam (HWSS-V), a profession-inclusive, culturally grounded instrument that extends the Health Professions Stress Inventory (HPSI) and the Nursing Stress Scale (NSS) by adding Vietnam-salient domains and crisis-monitoring utility.

**Methods:**

We conducted a cross-sectional survey of 520 physicians, nurses, and medical technicians at two university hospitals (June–December 2021). Fifty items adapted from HPSI/NSS underwent forward – backward translation and expert review. Psychometric evaluation included item-level content validity index (I-CVI), scale-level content validity index (S-CVI), exploratory factor analysis (EFA), confirmatory factor analysis (CFA), and reliability testing (Cronbach’s alpha).

**Results:**

All fifty items showed strong content validity (I-CVI ≥0.80; κ 0.67–0.97; S-CVI = 0.90). EFA supported a five-factor structure. After removing six low-loading items, forty-four items explained 87.1% of variance with excellent reliability (overall Cronbach’s alpha = 0.96; subscales 0.85–0.95). CFA indicated acceptable fit (Root Mean Square Error of Approximation = 0.077; Standardized Root Mean Squared Residual = 0.060; Tucker – Lewis Index = 0.827; Comparative Fit Index = 0.816).

**Conclusions:**

HWSS-V enables practical hospital-level stress surveillance and quality improvement. Hospitals can: (i) embed HWSS-V into biannual staff health checks to benchmark units and triage high-risk groups; (ii) integrate scores into dashboards to trigger tailored responses; and (iii) deploy rapid assessments during crises (e.g. outbreaks, patient surges) to guide resource allocation. By addressing culturally specific stressors across major clinical professions, HWSS-V provides actionable capabilities beyond HPSI/NSS for Vietnam’s hospitals.

## Paper Context

● **Main findings:** We developed and validated a forty-four-item Healthcare Worker Stress Scale-Vietnamese version with five domains and strong reliability, confirming a coherent factor structure in a sample of five hundred and twenty healthcare workers.● **Added knowledge:** The scale provides a culturally specific and profession-inclusive measure of healthcare worker stress, adding domains on work – family conflict and occupational hazards that are highly relevant in contemporary Vietnamese healthcare.● **Global health impact for policy and action:** The scale enables routine surveillance of staff stress and targeted organizational interventions, supporting workforce well-being and patient safety in resource-limited health systems.

## Background

Occupational stress among healthcare workers is a significant concern globally, given its implications for psychological well-being, job performance, and overall organizational health. Healthcare environments, characterized by demanding workloads, extended working hours, and emotional intensity, are widely recognized for their high-stress potential. In Vietnam, empirical studies highlight a substantial prevalence of occupational stress among healthcare professionals, with reported rates ranging from 7.7% to 44.6% [1,2]. Common stressors include excessive job demands, administrative pressures, limited control over work schedules, and emotionally challenging patient interactions. Prolonged exposure to these stressors can severely impact the mental health of healthcare workers, leading to outcomes such as burnout, reduced job satisfaction, and increased turnover intentions, all of which can compromise the quality of patient care [3,4]. The COVID-19 pandemic further exacerbated stress levels among Vietnamese healthcare staff, highlighting critical vulnerabilities related to staffing shortages, resource limitations, and the intense emotional strain of patient care during crises [5]. This experience underscored the importance of robust support systems and contextually appropriate tools for monitoring and managing the stress of healthcare workers during such emergencies, whether in future pandemics or other crises.

However, despite extensive research on occupational stress internationally, Vietnam currently lacks a context-specific, psychometrically validated instrument to measure occupational stress among its healthcare professionals. This absence of a tailored assessment tool represents a critical knowledge gap in addressing healthcare worker stress in the country. Current Vietnamese studies often rely on general psychological scales, such as the Depression, Anxiety, and Stress Scale-21, which do not fully capture the unique occupational stressors inherent in healthcare work [6–8]. Additionally, while adaptations of internationally recognized instruments, such as the Health Professions Stress Inventory (HPSI) and Nursing Stress Scale (NSS), have been utilized in Vietnam, these tools have not undergone rigorous cultural validation, raising concerns about their contextual relevance and accuracy [9,10]. This lack of a suitable measurement tool clearly indicates the need for a new, culturally tailored instrument to reliably assess occupational stress in Vietnam’s healthcare environment, given that existing measures are either too generic or not validated for this specific context.

Addressing this gap, the present study aimed to develop and validate the Healthcare Worker Stress Scale-Vietnamese (HWSS-V) through a systematic process of cross-cultural adaptation and psychometric evaluation. Drawing on established scales (HPSI and NSS) as a foundation, we undertook a comprehensive adaptation process with expert consultations to ensure cultural relevance and content validity. The resulting HWSS-V offers a much-needed, reliable tool for assessing occupational stress among Vietnamese healthcare workers. Not only will this instrument facilitate more accurate identification of stress levels and sources, but it will also provide a foundation for designing targeted interventions to enhance the well-being of healthcare workers, improve job performance, and safeguard patient safety in Vietnamese healthcare settings. Moreover, by enabling early identification of distress, the HWSS-V can help maintain healthcare workforce resilience during future emergencies.

## Methods

### Study design and setting

We conducted a cross-sectional study (June-December 2021) at two tertiary teaching hospitals in Vietnam: Hanoi Medical University Hospital and Thai Binh University of Medicine and Pharmacy Hospital. These hospitals were selected, representing a large urban academic center (Hanoi) and a provincial teaching hospital (Thai Binh). Both hospitals function as clinical training centers, providing patient care and engaging in research and technology transfer across various clinical departments. This choice ensured uniform research governance, high data quality, and access to diverse clinical departments. To mitigate limits on generalizability, we sampled across multiple departments and three professional groups (physicians, nurses, and medical technicians).

### Participants and recruitment

We employed consecutive convenience sampling, where all on-duty, eligible staff across departments were invited to participate during data collection windows. The site-level quotas were established by profession, including physicians, nurses, and medical technicians, to ensure adequate coverage across healthcare roles. Eligible participants were healthcare professionals with at least 6 months of work experience who provided informed consent. Those currently enrolled in full-time educational programs, receiving inpatient treatment, pregnant, or breastfeeding were excluded. Participants completed a self-administered Vietnamese questionnaire anonymously. No direct identifiers (e.g., name, phone number, email, employee ID, IP address) were collected, and responses were stored without linkage to personnel records. The final analytic sample comprised 520 respondents.

### Sample size justification

Based on an anticipated stress prevalence of 41.3% [11], 95% confidence level, and 11% relative error, the minimum sample size was 452; allowing for non-response, we aimed for a target of at least 520. This sample size satisfied common adequacy criteria for factor analysis (≥10 participants per item; *N* = 520 for 50 items), consistent with recommended ratios in the psychometrics literature [12–14].

### Measurements and instruments

#### Healthcare Worker Stress Scale-Vietnamese (HWSS-V)

Drawing on the Health Professions Stress Inventory (HPSI, 30 items) and the Nursing Stress Scale (NSS, 34 items) [9,10], we developed a 50-item self-report instrument capturing five domains: Work (13 items), Workplace Relationships (17), Relationships with Patients and Families (6), Work-Family Conflict (7), and Occupational Hazards (7). Items use a 5-point Likert scale (1 = never to 5 = very often); higher scores indicate greater stress *(see Supplementary 1–2).*

#### Covariates

##### Demographic characteristics

Participants provided demographic details, including age (in years), sex (male or female), marital status (living with spouse or single/divorced/separated), and educational level (vocational intermediate/college, university bachelor’s degree, or undergraduate).

##### Professional characteristics

Information on professional attributes included professional category (medical doctor, nurse, or medical technician), years of total work experience, and duration of employment at the current hospital (≤5 years, 5–10 years, 10–20 years, or >20 years).

### Translation and cross-cultural adaptation

Following standard cross-cultural adaptation procedures, two bilingual Vietnamese psychologists independently forward-translated selected HPSI/NSS items; an expert panel (physician, nurse, psychologist) reconciled discrepancies into a single Vietnamese draft. An independent bilingual translator performed back-translation to English, which was compared with the originals to ensure semantic and conceptual equivalence. Disagreements were resolved iteratively. Items were thematically organized; redundant content was merged; wording was refined for clinical relevance and clarity. A five-member expert panel (clinical psychology, community health, and nursing) assessed content validity and cultural appropriateness. Items were retained when expert agreement met the criteria of Item-Level Content Validity Index (I-CVI) ≥0.78–0.80 and Cohen’s Kappa (κ) ≥ 0.60. Items were modified when I-CVI scores ranged from 0.70 to 0.78 or when cognitive interviews revealed ambiguity, idioms, or setting-specific terminology that required clarification. Items were deleted or merged when I-CVI < 0.70, κ < 0.60, redundancy was evident, or the content lacked cultural applicability. The pre-final HWSS-V underwent pilot cognitive interviews with 20 healthcare workers, resulting in minor revisions to enhance readability and user experience.

### Statistical analysis

All statistical analyses were conducted using STATA 15.1 (StataCorp LP, College Station, USA) and RStudio. STATA 15.1 handled data management, descriptive statistics (means/standard deviations for continuous variables and frequencies/percentages for categorical variables), distributional checks (skewness, kurtosis), floor/ceiling flags ( >15%), item analyses (item – total and inter-item correlations), internal consistency (Cronbach’s α for total scale and domains), Exploratory Factor Analysis (EFA) for common-factor extraction with oblique rotation, Kaiser – Meyer – Olkin (KMO: measure of sampling adequacy with values > 0.80 indicate data are well-suited for factor analysis) and Bartlett’s test (tests whether the correlation matrix differs from identity, with significance indicates sufficient inter-item correlations for factor analysis). With confirmatory factor analysis (CFA), we specified an oblique five-factor measurement model and estimated it by maximum likelihood. We reported model chi-square with degrees of freedom (χ^2^ (df)), the normed chi-square (χ^2^/df), the Comparative Fit Index (CFI), the Tucker – Lewis Index (TLI), the Incremental Fit Index (IFI), the Normed Fit Index (NFI), the Root Mean Square Error of Approximation (RMSEA) with its 90% confidence interval, and the Standardized Root Mean Square Residual (SRMR). Latent factor correlations (φ) and subscale Cronbach’s alpha (α) were also presented. RStudio was used only to generate the PCA biplot (ggplot2/factoextra). Two-sided *p* < 0.05 was considered statistically significant.

#### Content validity

To establish the content validity of the questionnaire, the following indices were calculated from a panel of five experts from relevant fields (clinical psychology, community health, and nursing): (1) I-CVI, (2) Scale-Level Content Validity Index (S-CVI), and (3) κ [15].

- *I-CVI*: For each item, the I-CVI was computed using the formula I−CVI = A x N, where A is the number of experts who rated the item as 3 or 4 (i.e. ‘appropriate/clear’ or ‘very appropriate/clear’), and N (in this study) is 5, reflecting the total number of experts who participated in the evaluation [15,16].
- *S-CVI*: This index was calculated by averaging the I-CVI values across all items. I-CVI and S-CVI values of 0.8 or higher indicate satisfactory content validity at the item and scale levels, respectively [17].
- κ: Kappa was determined using the formula $\kappa =\;\left(I-\mathrm{CVI}\;-\;\mathrm{Pc}\right)\;x\;\left(1\;-\;\mathrm{Pc}\right)$, where Pc is the probability of correct selection by the experts, calculated as $\left[N!\;x\;A!\;x\;\left(N\;-\;A\right)!\right]\;x\;0.5\;x\;N$. κ value ≥0.6 is considered acceptable for indicating the reliability of items [18].

#### Reliability

Internal consistency was assessed using Cronbach’s alpha, with a value of 0.70 or higher indicating acceptable reliability [19]. In addition to calculating Cronbach’s alpha for the overall scale, we examined correlations among the various domains (domain-domain), correlations between individual items (item-item), correlations of each item with the total scale (item-total), and Cronbach’s alpha for each domain if specific items were removed. This approach provided a comprehensive understanding of how each item contributed to the overall reliability of the instrument.

#### Factorial structure

EFA, using a common factor extraction with oblique rotation, identified a factor structure. The number of factors was determined by examining the Scree plot, conducting a parallel analysis, and considering eigenvalues and the percentage of variance explained. Items with factor loadings of 0.40 or higher were retained in the corresponding components [20].

CFA was conducted to evaluate the factor structure of the HWSS-V identified by the exploratory analysis. For completeness, we also compared this primary model with more parsimonious one- and two-factor alternatives [21]. The overall fit of the measurement model was evaluated using RMSEA, SRMR, TLI, and CFI. Following widely used SEM guidelines, acceptable fit was defined as RMSEA < 0.08 [22] and SRMR < 0.08 [23]. Consistent with literature treating TLI/CFI ≥0.80 as a minimally acceptable (marginal) level of fit, while acknowledging that stricter thresholds (approximately 0.90–0.95) are often recommended for ‘good’ fit, we considered TLI ≥0.80 and CFI ≥0.80 as the lower bound for acceptability [23–25]. A two-sided *p* < 0.05 was considered statistically significant for all analyses.

Furthermore, we re-tested the measurement model via CFA using the finalized 44-item, five-factor configuration and reported a comprehensive set of fit indices: χ^2^ (df), χ^2^/df, CFI, TLI, IFI, NFI, RMSEA with 90% CI, and SRMR. We adopted commonly used benchmarks (CFI/TLI/NFI/IFI ≥0.90, RMSEA ≤0.08 with a narrow 90% CI, SRMR ≤0.08, and χ^2^/df ≈ 3–5) and, acknowledging the sample-size sensitivity of the chi-square test, emphasized approximate fit indices when judging overall model fit, consistent with recent psychometric development/adaptation studies that report CFA fit and construct-validity evidence using similar indices [26,27].

#### Convergent and divergent validity

A Pearson correlation matrix was generated to examine the convergent and divergent validity of the modified HWSS-V scale, focusing on correlations between individual items and their respective domains. Convergent validity was considered inadequate if the diagonal correlation values were below 0.40. Conversely, divergent validity was deemed inadequate if any off-diagonal correlation within the same row exceeded the corresponding diagonal value.

## Results

We analyzed 520 participants (mean age 34.6 ± 7.4 years); most were female, were medical doctors, and lived with a spouse. Around half held undergraduate degrees, and the mean work experience was 9.9 ± 6.7 years; few had ≥20 years at the current hospital (Table 1).

**Table 1.** Sociodemographic and professional characteristics of participants (*n* = 520).

Characteristics	Mean (SD)/Frequency (Percent)
**Sex**	
Male	208 (40.0%)
Female	312 (60.0%)
**Age** (years)	Mean = 34.6 (SD = 7.4)
**Professional categories**	
Medical doctor	242 (46.5%)
Nurse	211 (40.6%)
Medical technician	67 (12.9%)
**Marital status**	
Single/Divorced/separated	112 (21.5%)
Living with spouse	408 (78.5%)
**Education**	
Vocational/College	155 (29.8%)
Bachelor’s degree	132 (25.4%)
Postgraduate	233 (44.8%)
**Work experience at the current hospital (years)**	
≤5	163 (31.4%)
5-< 10	178 (34.2%)
10-< 20	153 (29.4%)
≥20	26 (5.0%)
**Work experience** (years)	Mean = 9.9 (SD = 6.7)

Content validity was strong (five experts): all 50 items had I-CVI ≥0.80 and S-CVI = 0.90; chance agreement was low (Pc = 0.03–0.16) and inter-rater agreement ranged from κ = 0.67–0.97 (Table 2).

**Table 2.** Content validity indices for the HWSS-V (*n* = 5 experts).

Items	A	I-CVI	Pc	κ	Items	A	I-CVI	Pc	κ
1	5	1.0	0.03	0.97	26	5	1.0	0.03	0.97
2	4	0.8	0.16	0.67	27	4	0.8	0.16	0.67
3	5	1.0	0.03	0.97	28	5	1.0	0.03	0.97
4	5	1.0	0.03	0.97	29	5	1.0	0.03	0.97
5	5	1.0	0.03	0.97	30	4	0.8	0.16	0.67
6	4	0.8	0.16	0.67	31	5	1.0	0.03	0.97
7	5	1.0	0.03	0.97	32	4	0.8	0.16	0.67
8	4	0.8	0.16	0.67	33	5	1.0	0.03	0.97
9	5	1.0	0.03	0.97	34	4	0.8	0.16	0.67
10	5	1.0	0.03	0.97	35	4	0.8	0.16	0.67
11	5	1.0	0.03	0.97	36	5	1.0	0.03	0.97
12	4	0.8	0.16	0.67	37	4	0.8	0.16	0.67
13	5	1.0	0.03	0.97	38	5	1.0	0.03	0.97
14	4	0.8	0.16	0.67	39	4	0.8	0.16	0.67
15	5	1.0	0.03	0.97	40	5	1.0	0.03	0.97
16	4	0.8	0.16	0.67	41	5	1.0	0.03	0.97
17	5	1.0	0.03	0.97	42	5	1.0	0.03	0.97
18	5	1.0	0.03	0.97	43	4	0.8	0.16	0.67
19	5	1.0	0.03	0.97	44	5	1.0	0.03	0.97
20	4	0.8	0.16	0.67	45	4	0.8	0.16	0.67
21	4	0.8	0.16	0.67	46	4	0.8	0.16	0.67
22	5	1.0	0.03	0.97	47	4	0.8	0.16	0.67
23	4	0.8	0.16	0.67	48	4	0.8	0.16	0.67
24	4	0.8	0.16	0.67	49	5	1.0	0.03	0.97
25	4	0.8	0.16	0.67	50	5	1.0	0.03	0.97
S-CVI	0.90								

A: Number of experts who rated the item as 3 or 4; I-CVI: Item Content Validity Index; S-CVI: Scale-Level Content Validity Index; Pc: Probability of chance agreement $\left(\mathrm{Pc}=\left[N!/\left(A!\left(N-A\right)!\right)\right]\times 0.5\text{\textbackslash{}\textasciicircum{}}N\right)$; **κ**: Cohen’s Kappa Coefficient $(\kappa =\left(I-\mathrm{CVI}-\mathrm{Pc}\right)/\left(1-\mathrm{Pc}\right)$.

**Figure 1.**
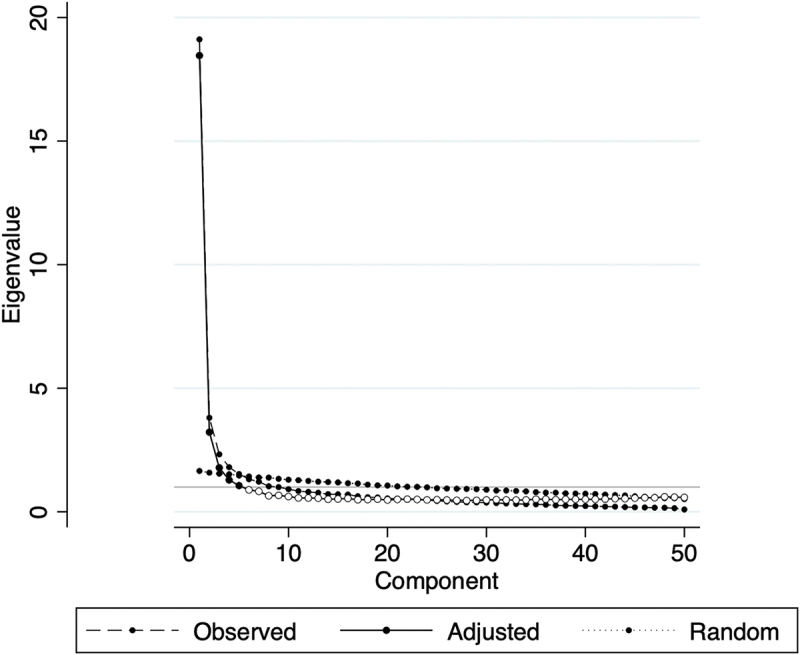
Scree and parallel-analysis plot (50 items).

Parallel analysis supported a five-factor solution (Figure 1). EFA showed sampling adequacy (KMO = 0.952) and a significant Bartlett’s test (χ^2^ = 17,906.27, df = 1,225, *p* < 0.001), indicating the correlations among items are strong enough and the data are suitable for factor analysis. The factors were labeled: (1) Workplace relationships (16 items), (2) Workload and job demands (12), (3) Occupational hazards (7), (4) Relationships with patients and families (6), and (5) Work – family conflict (5). Items with loadings <0.40 were removed (6 items), a standard criterion suggesting those items did not meaningfully align with any factor. Item Q43 cross-loaded on Factors 3 and 5 and was retained under Factor 5 based on the higher loading. The five factors explained 87.1% of the variance (Table 3).

The dashed curve shows observed eigenvalues from principal-component analysis; the solid curve shows mean random eigenvalues from Horn’s parallel analysis; the dotted curve shows 95th-percentile random eigenvalues. The first five observed eigenvalues exceed both reference curves, supporting a five-factor solution.

**Table 3.** EFA pattern matrix (oblimin-rotated) and communalities (h^2^) (*n* = 520).

Items	Factor 1	Factor 2	Factor 3	Factor 4	Factor 5	*h2*	Items	Factor 1	Factor 2	Factor 3	Factor 4	Factor 5	*h2*
q1		0.5680				0.4328	q26	0.7166					0.6268
q2		0.6844				0.5953	q27	0.7182					0.6265
q3		0.5566				0.4232	q28	0.7378					0.6286
q4		0.4659				0.3731	q29	0.6240					0.5216
q5						0.1124	q30	0.5229					0.4861
q6		0.4506				0.4264	q31				0.5592		0.5628
q7		0.5683				0.4094	q32				0.6369		0.5523
q8		0.5278				0.4661	q33				0.6794		0.7137
q9		0.5961				0.4872	q34				0.4481		0.4708
q10		0.6142				0.5897	q35				0.6488		0.6507
q11		0.4657				0.3534	q36				0.5524		0.5987
q12		0.6262				0.5996	q37					0.6092	0.7265
q13		0.6414				0.5811	q38					0.6884	0.8079
q14	0.4580					0.4403	q39					0.6725	0.7775
q15	0.6663					0.5389	q40						0.4971
q16	0.6216					0.4964	q41					0.4997	0.5376
q17	0.6581					0.5949	q42						0.5309
q18	0.7119					0.6422	q43			0.4910		0.4912	0.5253
q19	0.7304					0.6176	q44			0.6590			0.5294
q20	0.6695					0.5145	q45			0.5570			0.3935
q21	0.6862					0.5495	q46			0.6457			0.5548
q22	0.7178					0.6004	q47			0.6209			0.4982
q23	0.6500					0.5298	q48			0.6117			0.5323
q24	0.7103					0.6233	q49			0.6270			0.5252
q25						0.2965	q50			0.6936			0.5581

% of variance	Factor 1	Factor 2	Factor 3	Factor 4	Factor 5
30.5	18.4	17.1	10.9	10.2	

Factor 1: Workplace relationships; Factor 2: Workload and job demands; Factor 3: Occupational hazards; Factor 4: Relationships with patients and their families; Factor 5: Work – family conflict; Only factor loadings ≥0.40 are displayed; h^2^: Communality (h^2^).

**Figure 2.**
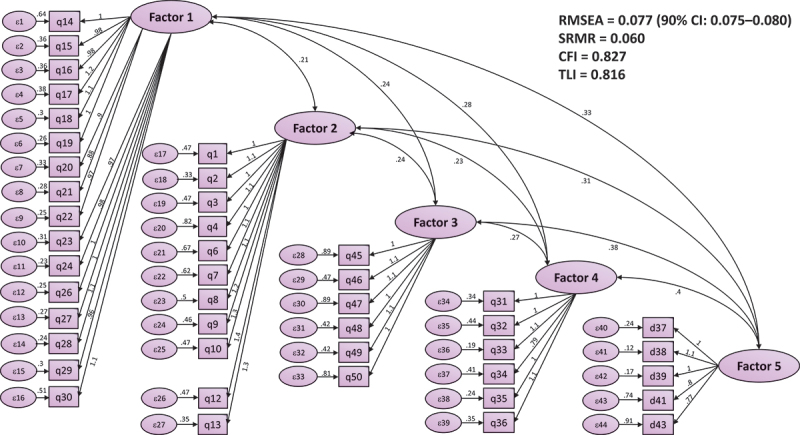
Confirmatory factor analysis (CFA) model for the 44-item healthcare worker stress scale-Vietnamese version (HWSS-V).

CFA and inter-factor associations. The final model (Figure 2) displayed model fit indices and the internal structure of the new HWSS-V. Several items were removed to achieve the model, which yielded goodness-of-fit indices of RMSEA = 0.077 (90% CI: 0.075–0.080), CFI = 0.827, TLI = 0.816, SRMR = 0.060, and a *p <*0.001. These results indicate a marginal yet acceptable fit: RMSEA and SRMR meet conventional cutoffs, whereas CFI and TLI are below 0.90 but meet the pre-specified minimal acceptability (≥0.80).

Laten factors: (F1) Workplace relationships; (F2) Workload/job demands; (F3) Occupational hazards; (F4) Patient – family relationships; (F5) Work – family conflict. Single-headed arrows indicate standardized loadings; double-headed arrows indicate inter-factor correlations. Model fit indices: RMSEA = 0.077 (90% CI 0.075–0.080), SRMR = 0.060, CFI = 0.827, TLI = 0.816.

Notes: (RMSEA: Root Mean Square Error of Approximation (model error per degree of freedom; ≤0.08 commonly acceptable). SRMR: Standardized Root Mean Squared Residual (≤0.08 commonly acceptable). CFI/TLI: Comparative Fit Index/Tucker – Lewis Index (≥0.90 often cited for ‘good’ fit; values in the high 0.80s are sometimes considered marginal/acceptable in early validation)).

For the 44 retained items (1–5 scale), distributions were acceptable (Skewness: −0.0 to 1.8; Kurtosis: 2.2 to 6.5), indicating no severe departures from normality. Item discrimination met conventional standards: corrected item – total correlations (r_it) were generally moderate to high (0.50–0.77), and α if item deleted did not suggest any removal that would materially improve domain reliability. Internal consistency was strong across all five factors (Cronbach’s alpha ≈0.86–0.95), with inter-item correlations (r) typically >0.50, supporting within-factor coherence (see Supplementary Table S3).

**Figure 3.**
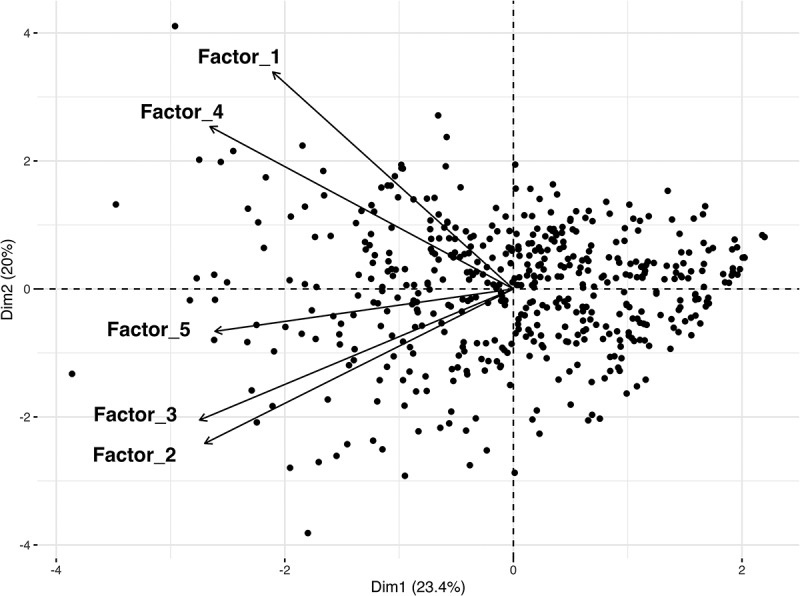
Biplot from principal components analysis of the item – factor structure.

Figure 3 presents the PCA biplot, illustrating how items align with the five domains. Arrows representing factor loadings revealed a strong correlation between Factors 1 and 4, while Factors 2, 3, and 5 contributed distinctively to the data structure. At the same time, those clustered near the origin exhibit a low correlation with both Dim1 and Dim2, indicating that these dimensions have less influence on them.

Dim1 explains 23.4% of the variance, and Dim2 explains 20.0% (total 43.4%). Arrows represent the direction and magnitude of the five factor-score vectors; points are standardized items. Items projecting strongly onto a vector align with that factor, whereas points clustered near the origin have weak associations with both plotted dimensions.

Notes: Dim1/Dim2: First and second principal components (percentage of variance explained shown in axis labels). PCA: Principal Components Analysis; biplot displays items (points) and factor-score vectors (arrows) in the same space.

We re-ran the CFA using the final 44-item configuration and estimated the oblique five-factor measurement model on the full sample (Table 4). All five subscales showed high internal consistency, with Cronbach’s α ranging from 0.86 to 0.95, indicating adequate stability of the latent constructs given the number of indicators per factor. CFA results yielding χ^2^ (891) = 3592.62, *p* < 0.001 and χ^2^/df = 4.03. Approximate fit indices indicated acceptable fit for an initial validation: RMSEA = 0.076 (90% CI: 0.074–0.079) and SRMR = 0.061. Incremental fit indices were marginal at this stage (CFI = 0.826; TLI = 0.826; IFI = 0.826; NFI = 0.781). The latent factor correlation matrix (φ) showed that all inter-factor correlations were <0.85, supporting discriminant validity while reflecting the expected, moderate relatedness among stress domains. Overall, the reliability, model fit, and pattern of latent correlations support an acceptable measurement model for this initial validation of the scale.

**Table 4.** Subscale reliability, confirmatory factor analysis (CFA) – measurement model: global fit indices & correlation matrix (φ) from CFA (*n* = 520).

Subscale reliability					Cronbach’s α
Factor 1					0.95
Factor 2					0.90
Factor 3					0.86
Factor 4					0.89
Factor 5					0.89
Model fit indices					Value
χ^2^ (df)					3592.62 (891)
χ^2^/df					4.03
CFI					0.826
TLI					0.826
IFI					0.826
NFI					0.781
RMSEA (90% CI)					0.076 (0.074–0.079)
SRMR					0.061
*p*					<0.001
	Factor 1	Factor 2	Factor 3	Factor 4	Factor 5
Factor 1	1.000	0.628	0.580	0.730	0.644
Factor 2	0.628	1.000	0.637	0.659	0.686
Factor 3	0.580	0.637	1.000	0.595	0.627
Factor 4	0.730	0.659	0.595	1.000	0.713
Factor 5	0.644	0.686	0.627	0.713	1.000

χ^2^ (df): Model chi-square with degrees of freedom (overall discrepancy from the saturated model; lower is better). χ^2^/df: Normed chi-square (≤3–5 often considered acceptable). CFI: Comparative Fit Index (≥0.90 often cited for ‘good’ fit; high-0.80s viewed as acceptable). TLI: Tucker – Lewis Index (≥0.90 commonly acceptable). IFI: Incremental Fit Index (≥0.90 commonly acceptable). NFI: Normed Fit Index (≥0.90 commonly acceptable). RMSEA (90% CI): Root Mean Square Error of Approximation with 90% confidence interval (≤0.08 commonly acceptable; ≤0.05 indicates close fit). SRMR: Standardized Root Mean Square Residual (≤0.08 commonly acceptable). p: *p*-value for the chi-square test (*p* < 0.05 indicates the model fits). Entries are latent factor correlations (φ) estimated from the CFA; diagonal elements are 1.00 by definition. All inter-factor correlations are below 0.85, supporting acceptable discriminant validity.

**Figure 4.**
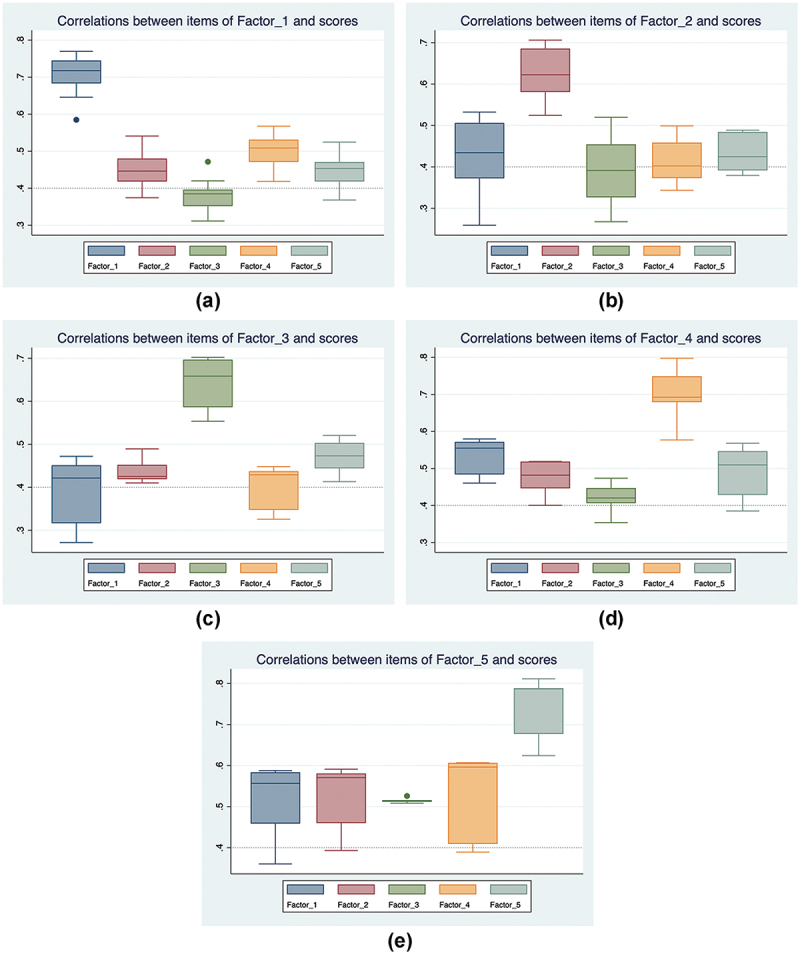
Correlations between items within each domain and scores of other domains.

All 44 items correlated ≥0.40 with their intended domains (convergent validity) and more strongly with their own factor than with others (discriminant validity), indicating each item measures its target domain better than alternative domains (Table 5). Figure 4 further visualises this through boxplots, confirming that items correlate more strongly within their domain and less so with others.

Each panel summarizes the distribution of correlations between items belonging to one factor and the scores of that factor and the other four factors. Boxes show the interquartile range; center lines mark medians; whiskers extend to 1.5×IQR; dots denote outliers. Correlations are Pearson’s r (*n* = 520).

**Table 5.** Convergent and divergent (discriminant) validities through the correlation matrix (*n* = 520).

Items	Factor 1	Factor 2	Factor 3	Factor 4	Factor 5	Items	Factor 1	Factor 2	Factor 3	Factor 4	Factor 5
q14	0.585	0.541	0.395	0.462	0.472	q8	0.532	0.623	0.363	0.429	0.388
q15	0.688	0.467	0.311	0.418	0.368	q9	0.372	0.638	0.431	0.400	0.451
q16	0.680	0.469	0.385	0.476	0.423	q10	0.421	0.667	0.503	0.459	0.425
q17	0.746	0.519	0.384	0.502	0.501	q12	0.470	0.686	0.520	0.476	0.484
q18	0.762	0.498	0.405	0.511	0.520	q13	0.522	0.697	0.449	0.450	0.489
q19	0.744	0.445	0.377	0.466	0.464	q45	0.316	0.410	0.554	0.326	0.413
q20	0.669	0.406	0.355	0.457	0.389	q46	0.439	0.452	0.697	0.436	0.520
q21	0.686	0.419	0.387	0.476	0.410	q47	0.404	0.424	0.654	0.422	0.444
q22	0.741	0.418	0.392	0.507	0.413	q48	0.472	0.489	0.663	0.448	0.498
q23	0.696	0.451	0.332	0.515	0.448	q49	0.451	0.419	0.702	0.437	0.503
q24	0.748	0.448	0.398	0.568	0.429	q50	0.272	0.427	0.586	0.347	0.448
q26	0.740	0.391	0.349	0.543	0.431	q31	0.544	0.509	0.406	0.697	0.529
q27	0.735	0.374	0.348	0.538	0.459	q32	0.460	0.446	0.354	0.679	0.429
q28	0.770	0.424	0.361	0.525	0.459	q33	0.580	0.519	0.447	0.798	0.547
q29	0.701	0.437	0.420	0.510	0.469	q34	0.484	0.401	0.474	0.577	0.385
q30	0.646	0.491	0.472	0.538	0.525	q35	0.572	0.455	0.424	0.749	0.490
q1	0.373	0.596	0.310	0.373	0.433	q36	0.566	0.519	0.417	0.688	0.568
q2	0.449	0.706	0.392	0.389	0.488	q37	0.588	0.591	0.526	0.596	0.788
q3	0.434	0.607	0.327	0.402	0.379	q38	0.584	0.581	0.515	0.607	0.811
q4	0.259	0.524	0.454	0.344	0.392	q39	0.557	0.571	0.515	0.607	0.788
q6	0.506	0.527	0.268	0.499	0.393	q41	0.459	0.460	0.512	0.409	0.677
q7	0.340	0.581	0.355	0.355	0.414	q43	0.360	0.393	0.509	0.389	0.624

Coefficients are item–factor correlations; values ≥0.400 indicate convergent validity.

## Discussion

### Principal findings

In this study, we developed and validated the HWSS‐V, adapted from two established instruments, the HPSI [9] and NSS [10]. Our psychometric evaluation showed that the HWSS‐V possesses sound measurement properties, including strong content validity, good internal consistency, a coherent factor structure, and satisfactory convergent and divergent validity. These findings underscore the instrument’s potential utility for assessing occupational stress among healthcare professionals in Vietnam.

### Cross-cultural adaptation and factor structure

In line with best-practice guidelines for cross-cultural adaptation, our forward-backwards translation process and expert panel reviews ensured that all 50 initial items were contextually relevant and linguistically precise [28,29]. Regarding content validity, the HWSS-V underwent rigorous evaluation, with all items surpassing the accepted I-CVI threshold of 0.80 and an overall S-CVI of 0.90, demonstrating strong expert agreement on item relevance [30]. Exploratory factor analysis yielded a five-factor solution: (1) Workplace Relationships, (2) Workload and Job Demands, (3) Occupational Hazards, (4) Relationships with Patients and Their Families, and (5) Work-Family Conflict. This structure encompasses core domains from NSS and HPSI, although organized somewhat differently. The NSS originally comprised seven domains (stress from workload, conflicts with physicians and other nurses, insufficient preparation, lack of support, patient death/dying, and uncertainty in treatment) [10], while HPSI included four broader domains (workload/responsibility, lack of recognition, interpersonal conflict, and professional uncertainty). The HWSS-V thus represents a balanced integration of these domains. For instance, Workplace Relationships combine interpersonal stressors from NSS (‘conflicts with physicians,’ ‘conflicts with other nurses’) into one factor, aligning closely with interpersonal conflicts in the HPSI. Similarly, Workload and Job Demands reflect heavy workloads noted across both original scales [9,10,31–33]. Domains such as ‘insufficient preparation’ or ‘uncertainty’ from the NSS merged into broader factors, reflecting the combined stress experiences of Vietnamese healthcare workers. Overall, comparable domains indicate that the HWSS-V’s factor structure is coherent and consistent with international occupational stress patterns. Confirmatory factor analysis supported this coherence, and high inter-item correlations within each factor further affirmed the unified dimension of each factor.

### Reliability and construct validity

On internal consistency, the HWSS-V showed excellent reliability. Cronbach’s alpha for the total scale was very high (likely ≥0.95). These values are comparable to or higher than those reported for the HPSI and NSS in prior studies. For instance, the original NSS demonstrated strong internal consistency, with a Cronbach’s alpha of approximately 0.89 in a Spanish sample [34]. Subsequent validations among Chinese nurses in Taiwan reported a similarly high Cronbach’s alpha of roughly 0.91 for the total NSS scale [35]. Likewise, the HPSI demonstrated good reliability, with a Cronbach’s alpha of approximately 0.88 in a study among primary care doctors in Malaysia [36]. Thus, the HWSS-V’s internal consistency meets the benchmarks set by established instruments, suggesting that its items cohesively measure the intended construct of work stress. Additionally, item-total correlations in the HWSS-V were all above 0.40, with each item correlating more strongly with its own subscale than with others. This indicates good convergent validity for each item with its domain and solid divergent (discriminant) validity between different stress domains. Such clarity of item-domain alignment is comparable to evidence from other scale evaluations (e.g. the Spanish version of the NSS demonstrated distinct subscale correlations with external criteria, supporting the discriminant validity of each) [34]. In summary, the HWSS-V’s reliability and internal validity metrics suggest that it is as psychometrically sound as the HPSI and NSS, while providing a more contextually relevant tool for Vietnam.

The development and validation of the HWSS-V align with findings from similar stress scales adapted in other countries, such as China, Indonesia, and Brazil. Cross-cultural validation studies of healthcare stress instruments consistently emphasise the importance of linguistic equivalence and contextual relevance, a principle we rigorously applied in creating the HWSS-V. For instance, the Chinese version of the NSS (translated for use in Taiwan) underwent a careful translation review, achieving approximately 92% meaning equivalence with the original English version [35]. The Chinese NSS demonstrated an overall Cronbach’s α of 0.91 (with subscales ranging from 0.67 to 0.79) and explained 53.77% of the variance, indicating that the core structure of the NSS was retained mainly in the Chinese version [35]. Our HWSS-V demonstrates similarly high internal consistency and variance explanation (totaling around 87.1% in EFA), comparable to those international findings, suggesting that the fundamental construct of healthcare work stress is transferable across cultures.

### Theoretical contribution and Vietnam-specific insights

Anchored in the Job Demands-Resources (JD-R) theory and Person-environment fit (P-E fit) framework [37,38], our findings clarify how context-bound stressors organize for Vietnamese healthcare workers. Two factors, Work-Family Conflict and Occupational Hazards, emerged as distinct, high-salience domains that (i) extend prevailing stressor taxonomies developed large in high-income contexts and (ii) align with sociocultural features of Vietnam (collectivism, hierarchical teams, and strong filial obligations) [39–41]. By empirically separating hazard-related risks (e.g. infection, aggression) from generic workload/interpersonal demands, the HWSS-V foregrounds threats that are structurally persistent in resource-constrained hospitals and post-pandemic care. Likewise, distinguishing work – family tensions highlights culturally specific spillover of extended caregiving roles into clinical work. Together, these insights refine theory by specifying which ‘demands’ are most consequential in an LMIC health system and motivate future tests of measurement invariance across professional groups and facility tiers.

### Added value and contextual innovations

The HWSS-V is not merely a translation, but a purpose-built tool tailored to the Vietnamese healthcare context. It integrates items from the HPSI and NSS into five parsimonious, culturally resonant factors [9,10]; and introduces two high-salience domains, Work – Family Conflict and Occupational Hazards, capturing caregiving pressures and safety risks (e.g. infection, aggression) that are especially salient in post-COVID and resource-limited hospitals. Interpersonal stressors are consolidated into a single Workplace Relationships factor, aligning with hierarchical/harmony norms. Unlike the nurse-specific NSS, the HWSS-V is profession-inclusive (encompassing nurses, physicians, and technicians), facilitating routine surveillance, cross-unit benchmarking, and targeted responses. Overall, it offers a culturally informed, contemporary measure of healthcare worker stress, featuring a streamlined structure and strong applicability in Vietnamese healthcare systems.

### Policy and practice implications

The successful validation of the HWSS-V has direct, actionable implications for Vietnam’s health system and comparable LMIC settings. It offers hospital administrators, occupational-health teams, and researchers a reliable, culturally attuned instrument to quantify work-related stress, addressing a long-standing reliance on foreign or ad hoc tools. When embedded in routine staff health checks (e.g. quarterly or biannual), HWSS-V scores can flag at-risk units early and trigger tiered responses (unit-level feedback dashboards; schedule flexibility and peer support for moderate stress; structured psychosocial programs and managerial coaching for high stress; and organizational fixes (staffing, workload redistribution, role clarity) where unit-level stress is elevated). Aggregated results can inform hospital accreditation metrics and national occupational-health standards.

### Regional applicability to Southeast Asia

The HWSS-V is highly portable across Southeast Asia, where health systems share similar features: collectivist work norms, hierarchical team structures, chronic workload pressures, and prevalent occupational hazards. We outline a pragmatic adoption pathway: (1) forward – backward translation and cognitive debriefing with mixed cadres; (2) pilot testing with configural/metric/scalar invariance to ensure score comparability; (3) calibration of percentile-based thresholds for local actionability; and (4) integration into quality-improvement cycles (quarterly dashboards, unit action plans). This pathway enables cross-profession and cross-country benchmarking while respecting local specificity. Open access to items/procedures further supports harmonized implementation and regional research collaborations.

### Limitations and future directions

While the HWSS-V demonstrates reliable psychometric properties, several limitations warrant consideration. First, the cross-sectional, single-occasion design precluded the assessment of temporal stability; therefore, we could not estimate test-retest reliability. Future studies should incorporate short-interval retesting (2–4 weeks) to calculate intraclass correlation coefficients and establish stability for individual-level monitoring. In addition, longitudinal designs are needed to examine predictive validity, for example, whether HWSS-V scores forecast subsequent burnout, sickness absence, turnover intention, unit-level quality indicators, or adverse events. Second, we did not administer an external comparator instrument; therefore, concurrent validity could not be evaluated in this study. Future studies should correlate HWSS-V scores with conceptually related measures (e.g. DASS-21 stress subscale, HPSI/NSS where culturally validated) to further establish criterion-related validity. Third, our sample came from two northern tertiary teaching hospitals with academic cultures, strong referral roles, and relatively better staffing and training resources. Such contexts can shape stress salience (e.g. heavier teaching – research demands, complex casemix, hierarchical team structures) compared with provincial/private facilities or primary care, where resource scarcities, multitasking, and patient volume patterns differ. Consequently, external validity may be limited, and future studies should recruit across regions (North – Central – South), ownership types (public/private), and care levels (primary – tertiary) with stratified sampling and site weights to derive nationally representative norms. Fourth, reliance on self-administered questionnaires may introduce self-report and social-desirability bias, potentially underestimating sensitive stressors (e.g. interpersonal conflict). Incorporating qualitative interviews or objective measures such as absenteeism records or physiological biomarkers could enhance construct validity. Mixed-methods designs and objective indicators (e.g. absenteeism records, error reports, and physiological markers) could enhance construct validity. Fifth, although the CFI/TLI indices were below 0.90, they met our a priori minimal threshold (≥0.80); the RMSEA/SRMR values were acceptable, and the factor solution was theoretically coherent. This model will serve as a target for future refinements and multi-site validations, marking a point for improvement. Finally, the present study was not powered to rigorously test between-profession differences (physicians vs. nurses vs. technicians). Future research should evaluate measurement invariance (configural/metric/scalar) and potential differential item functioning across professional groups and settings. If supported, it should develop profession-specific norms and actionable thresholds to guide tailored interventions. Collectively, these steps will strengthen the evidence base for deploying the HWSS-V in routine surveillance and quality-improvement cycles at scale.

## Conclusions

The HWSS-V is a reliable and valid instrument for assessing work-related stress among Vietnamese healthcare workers. Its five empirically derived domains encompass both universal and context-specific stressors. Beyond research use, the HWSS-V is suitable for routine administration within healthcare systems to monitor stress at the individual, unit, and hospital levels, track trends over time, identify groups with elevated stress for timely support, and inform targeted interventions and policy adjustments as part of occupational health and quality improvement cycles. By enabling regular, comparable reporting across professional groups and departments, the HWSS-V can help hospitals evaluate the impact of staffing, workflow, and well-being initiatives and guide efforts to reduce burnout.

## Supplementary Material

STROBE_checklist.pdf

Supplementary_Materials_clean.docx

## Data Availability

De-identified data underlying this article are available from the corresponding author on reasonable request. Because the dataset contains potentially identifying information about healthcare professionals, public sharing is not permitted under the ethics approval issued by the Hanoi Medical University Institutional Ethical Review Board, approval No. 400/GCN-HDDDNCYSH-ĐHYHN. Requests will be considered by the study team, and a data-use agreement and additional ethics clearance may be required.
