# Supplementary material for: Development and validation of the Healthcare Worker Stress Scale-Vietnamese: a culturally grounded instrument to assess work-related stress
Source: Glob Health Action. 2025 Oct 31;18(1):2576369. doi: 10.1080/16549716.2025.2576369 (PMC12581766; doi:10.1080/16549716.2025.2576369)
Supplement: Supplementary_Materials_clean.docx [file ZGHA_A_2576369_SM0672.docx]

**SUPPLEMENTARY INFORMATION**

These supplementary files includes the full English and Vietnamese versions of the Healthcare Worker Stress Scale–Vietnam (HWSS-V), developed and validated in this study. The tool consists of 50 items across five domains, with 5-point Likert-scale responses.

**Supplementary Table 1 Contents of the Healthcare Worker Stress Scale–Vietnam (English version).**

Please indicate how frequently each event or condition applies to you by selecting the most appropriate response using the following scale:

1 – Never (These events never occur)

2 – Rarely (These events occur and persist about 25% of the time)

3 – Occasionally (These events occur and persist about 50% of the time)

4 – Often (These events occur and persist about 75% of the time)

5 – Very Often (These events occur and persist almost constantly)

| **No** | **Item** | **Response** | | | | |
| --- | --- | --- | --- | --- | --- | --- |
|  |  | **1** | **2** | **3** | **4** | **5** |
| **I. Workload and job demands** | | | | | | |
| q1 | Lack of time for adequate rest | 1 | 2 | 3 | 4 | 5 |
| q2 | Excessive workload beyond personal capacity | 1 | 2 | 3 | 4 | 5 |
| q3 | Poor work organization and arrangement | 1 | 2 | 3 | 4 | 5 |
| q4 | High-risk job | 1 | 2 | 3 | 4 | 5 |
| q5 | Job characteristics involve little interaction with people | 1 | 2 | 3 | 4 | 5 |
| q6 | Fear of making mistakes in patient treatment | 1 | 2 | 3 | 4 | 5 |
| q7 | Working at high intensity to meet deadlines | 1 | 2 | 3 | 4 | 5 |
| q8 | Unclear job assignments | 1 | 2 | 3 | 4 | 5 |
| q9 | Staff shortage | 1 | 2 | 3 | 4 | 5 |
| q10 | Managing (monitoring/examining) too many patients | 1 | 2 | 3 | 4 | 5 |
| q11 | Work requires high responsibility | 1 | 2 | 3 | 4 | 5 |
| q12 | Constantly feeling overwhelmed by patient overload | 1 | 2 | 3 | 4 | 5 |
| q13 | Insufficient time to complete assigned work | 1 | 2 | 3 | 4 | 5 |
| **II. Workplace relationships** | | | | | | |
| q14 | Fear of displeasing superiors | 1 | 2 | 3 | 4 | 5 |
| q15 | Difficulty establishing close relationships with colleagues | 1 | 2 | 3 | 4 | 5 |
| q16 | Team members are not available to assist in urgent situations | 1 | 2 | 3 | 4 | 5 |
| q17 | Difficulty communicating with superiors about work | 1 | 2 | 3 | 4 | 5 |
| q18 | Lack of support from superiors at work | 1 | 2 | 3 | 4 | 5 |
| q19 | Lack of support from colleagues at work | 1 | 2 | 3 | 4 | 5 |
| q20 | Psychological abuse: threats, discrimination, bullying, harassment | 1 | 2 | 3 | 4 | 5 |
| q21 | Verbal abuse, including insults and sarcastic comments | 1 | 2 | 3 | 4 | 5 |
| q22 | Conflicts with doctors (or colleagues) | 1 | 2 | 3 | 4 | 5 |
| q23 | Constant criticism from superiors | 1 | 2 | 3 | 4 | 5 |
| q24 | Lack of team support affects patient trust | 1 | 2 | 3 | 4 | 5 |
| q25 | Insufficient equipment for patient care | 1 | 2 | 3 | 4 | 5 |
| q26 | No opportunity to openly discuss work issues with colleagues | 1 | 2 | 3 | 4 | 5 |
| q27 | No opportunity to share experiences and emotions with colleagues | 1 | 2 | 3 | 4 | 5 |
| q28 | Difficulty working with colleagues | 1 | 2 | 3 | 4 | 5 |
| q29 | Work-related conflicts with colleagues | 1 | 2 | 3 | 4 | 5 |
| q30 | Limited access to directly discuss department issues with leadership | 1 | 2 | 3 | 4 | 5 |
| **III. Relationships with patients and their families** | | | | | | |
| q31 | Feeling helpless when a patient's condition does not improve | 1 | 2 | 3 | 4 | 5 |
| q32 | Feeling helpless when witnessing a patient’s death | 1 | 2 | 3 | 4 | 5 |
| q33 | Feeling unprepared to support the emotions of a patient’s family | 1 | 2 | 3 | 4 | 5 |
| q34 | Facing negative reactions from patients or their families | 1 | 2 | 3 | 4 | 5 |
| q35 | Uncertainty about what to say to a patient's family regarding their condition and treatment | 1 | 2 | 3 | 4 | 5 |
| q36 | Insufficient time to provide emotional support to patients | 1 | 2 | 3 | 4 | 5 |
| **IV. Work–family conflict** | | | | | | |
| q37 | Work burden affecting family life | 1 | 2 | 3 | 4 | 5 |
| q38 | Excessive working hours making it difficult to fulfill family responsibilities | 1 | 2 | 3 | 4 | 5 |
| q39 | Work pressure making it hard to meet family obligations | 1 | 2 | 3 | 4 | 5 |
| q40 | Difficulty taking leave in case of family emergencies | 1 | 2 | 3 | 4 | 5 |
| q41 | Financial difficulties in the family | 1 | 2 | 3 | 4 | 5 |
| q42 | Spouse's or children's health issues affecting work | 1 | 2 | 3 | 4 | 5 |
| q43 | Need to find additional work to ensure financial stability | 1 | 2 | 3 | 4 | 5 |
| **V. Occupational hazards** | | | | | | |
| q44 | Working in an environment with a high risk of infectious diseases | 1 | 2 | 3 | 4 | 5 |
| q45 | Exposure to radiation or strong light sources such as X-rays, UV rays, or lasers | 1 | 2 | 3 | 4 | 5 |
| q46 | Workplace does not provide sufficient protective equipment | 1 | 2 | 3 | 4 | 5 |
| q47 | Working in excessively hot conditions | 1 | 2 | 3 | 4 | 5 |
| q48 | Noisy and chaotic workplace environment | 1 | 2 | 3 | 4 | 5 |
| q49 | Poor workplace lighting (too dim or too bright) | 1 | 2 | 3 | 4 | 5 |
| q50 | High risk of infection at the workplace | 1 | 2 | 3 | 4 | 5 |

**Supplementary Table 2 Contents of the Healthcare Worker Stress Scale–Vietnam (Vietnamese version).**

Xin anh/chị hãy điền vào ô phù hợp nhất với bản thân với các số tương ứng với các mức độ

1= Không bao giờ (các sự kiện không xảy ra bao giờ)

2= Hiếm khi (các sự kiện xảy ra và tồn tại khoảng 25% thời gian)

3= Thỉnh thoảng (các sự kiện xảy ra và tồn tại khoảng 50% thời gian)

4= Thường xuyên (các sự kiện xảy ra và tồn tại khoảng 75% thời gian)

5= Rất thường xuyên (các sự kiện xảy ra và luôn tồn tại)

| **TT** | **Nội dung** | **Mức độ** | | | | |
| --- | --- | --- | --- | --- | --- | --- |
|  |  | 1 | 2 | 3 | 4 | 5 |
| **I. Khối lượng công việc và yêu cầu công việc** | | | | | | |
| 1 | Không có thời gian để nghỉ ngơi đầy đủ | 1 | 2 | 3 | 4 | 5 |
| 2 | Khối lượng công việc quá nhiều vượt quá khả năng làm việc của bản thân | 1 | 2 | 3 | 4 | 5 |
| 3 | Tổ chức sắp xếp công việc chưa hợp lý | 1 | 2 | 3 | 4 | 5 |
| 4 | Công việc có độ nguy hiểm cao | 1 | 2 | 3 | 4 | 5 |
| 5 | Đặc điểm công việc ít giao tiếp với mọi người | 1 | 2 | 3 | 4 | 5 |
| 6 | Sợ sai sót trong điều trị người bệnh | 1 | 2 | 3 | 4 | 5 |
| 7 | Làm việc với cường độ cao để hoàn thành công việc đúng thời gian được giao | 1 | 2 | 3 | 4 | 5 |
| 8 | Sự phân công công việc không rõ ràng | 1 | 2 | 3 | 4 | 5 |
| 9 | Tình trạng thiếu nhân lực | 1 | 2 | 3 | 4 | 5 |
| 10 | Chăm sóc (quản lý/khám) quá nhiều bệnh nhân | 1 | 2 | 3 | 4 | 5 |
| 11 | Công việc đòi hỏi nhiều trách nhiệm | 1 | 2 | 3 | 4 | 5 |
| 12 | Luôn bị ám ảnh bởi tình trạng bệnh nhân quá tải | 1 | 2 | 3 | 4 | 5 |
| 13 | Không đủ thời gian để hoàn thành công việc của mình | 1 | 2 | 3 | 4 | 5 |
| **II. Mối quan hệ tại nơi làm việc** | | | | | | |
| 14 | Sợ làm mất lòng cấp trên | 1 | 2 | 3 | 4 | 5 |
| 15 | Không tạo được mối quan hệ thân thiết với đồng nghiệp | 1 | 2 | 3 | 4 | 5 |
| 16 | Các thành viên trong nhóm không kịp xuất hiện để giúp đỡ kịp thời trong những trường hợp cấp bách | 1 | 2 | 3 | 4 | 5 |
| 17 | Cảm thấy khó nói chuyện với cấp trên về công việc | 1 | 2 | 3 | 4 | 5 |
| 18 | Ít nhận được sự hỗ trợ từ cấp trên khi làm việc | 1 | 2 | 3 | 4 | 5 |
| 19 | Ít nhận được sự hỗ trợ khi làm việc của đồng nghiệp | 1 | 2 | 3 | 4 | 5 |
| 20 | Bị lạm dụng tâm lý như đe dọa, phân biệt đối xử, bắt nạt và quấy rối | 1 | 2 | 3 | 4 | 5 |
| 21 | Bị lạm dụng lời nói như lăng mạ, bình luận mỉa mai | 1 | 2 | 3 | 4 | 5 |
| 22 | Xung đột với bác sĩ (hoặc đồng nghiệp) | 1 | 2 | 3 | 4 | 5 |
| 23 | Luôn bị chỉ trích từ cấp trên | 1 | 2 | 3 | 4 | 5 |
| 24 | Thiếu sự hỗ trợ từ ê-kíp cùng làm việc ảnh hưởng đến lòng tin của bệnh nhân | 1 | 2 | 3 | 4 | 5 |
| 25 | Thiếu trang thiết bị phục vụ bệnh nhân | 1 | 2 | 3 | 4 | 5 |
| 26 | Không có cơ hội để nói chuyện cởi mở với đồng nghiệp trong công việc | 1 | 2 | 3 | 4 | 5 |
| 27 | Không có cơ hội để chia sẻ kinh nghiệm và cảm xúc với đồng nghiệp | 1 | 2 | 3 | 4 | 5 |
| 28 | Gặp khó khăn khi làm việc chung với đồng nghiệp | 1 | 2 | 3 | 4 | 5 |
| 29 | Bất đồng với đồng nghiệp liên quan đến công việc | 1 | 2 | 3 | 4 | 5 |
| 30 | Thiếu tiếp cận để nói thẳng thắn với lãnh đạo về những vấn đề ở khoa/ phòng | 1 | 2 | 3 | 4 | 5 |
| **III. Mối quan hệ với bệnh nhân và người nhà bệnh nhân** | | | | | | |
| 31 | Cảm thấy bất lực khi thấy tình trạng bệnh của bệnh nhân không cải thiện | 1 | 2 | 3 | 4 | 5 |
| 32 | Cảm thấy bất lực khi chứng kiến cái chết của một bệnh nhân | 1 | 2 | 3 | 4 | 5 |
| 33 | Cảm giác thiếu sự chuẩn bị để giúp đỡ với những cảm xúc của gia đình người bệnh | 1 | 2 | 3 | 4 | 5 |
| 34 | Bị bệnh nhân/ người nhà bệnh nhân phản ứng không tốt: chửi mắng, đe dọa, hành hung | 1 | 2 | 3 | 4 | 5 |
| 35 | Không biết phải nói thế nào với người nhà bệnh nhân về tình trạng sức khỏe và việc điều trị | 1 | 2 | 3 | 4 | 5 |
| 36 | Không đủ thời gian để hỗ trợ tinh thần cho bệnh nhân | 1 | 2 | 3 | 4 | 5 |
| **IV. Xung đột giữa công việc và gia đình** | | | | | | |
| 37 | Gánh nặng công việc ảnh hưởng đến cuộc sống gia đình | 1 | 2 | 3 | 4 | 5 |
| 38 | Thời gian công việc nhiều khiến bản thân khó có thể hoàn thành nhiệm vụ gia đình | 1 | 2 | 3 | 4 | 5 |
| 39 | Áp lực công việc khiến bản thân khó hoàn thành nghĩa vụ gia đình | 1 | 2 | 3 | 4 | 5 |
| 40 | Khó xin nghỉ phép trong trường hợp gia đình có việc khẩn cấp | 1 | 2 | 3 | 4 | 5 |
| 41 | Kinh tế tiền bạc gia đình thiếu hụt | 1 | 2 | 3 | 4 | 5 |
| 42 | Vấn đề sức khỏe của vợ/chồng, con cái ảnh hưởng đến công việc | 1 | 2 | 3 | 4 | 5 |
| 43 | Phải nghĩ cách làm thêm để đảm bảo thu nhập | 1 | 2 | 3 | 4 | 5 |
| **V. Mối nguy hiểm nghề nghiệp** | | | | | | |
| 44 | Làm việc trong môi trường có nguy cơ lây nhiễm bệnh từ nơi làm việc | 1 | 2 | 3 | 4 | 5 |
| 45 | Tiếp xúc với bức xạ hoặc ánh sáng mạnh như tia X, tia cực tím, tia laser | 1 | 2 | 3 | 4 | 5 |
| 46 | Nơi làm việc không cung cấp đủ thiết bị bảo hộ lao động | 1 | 2 | 3 | 4 | 5 |
| 47 | Nơi làm việc quá nóng | 1 | 2 | 3 | 4 | 5 |
| 48 | Nơi làm việc ồn ào, lộn xộn | 1 | 2 | 3 | 4 | 5 |
| 49 | Ánh sáng nơi làm việc quá yếu hoặc quá mạnh | 1 | 2 | 3 | 4 | 5 |
| 50 | Nơi làm việc dễ nhiễm bệnh | 1 | 2 | 3 | 4 | 5 |

**Supplementary Table 3. Basic descriptions and reliability of the HWSS-V (n = 520)**

| **Items** | **Responses (%)** | | | | | | **Mean (SD)** | | **Skewness** | | **Kurtosis** | | **Floor (%)** | **Ceiling (%)** | **Item total correlation** | | **Cronbach’s alpha if item deleted** |  |
| --- | --- | --- | --- | --- | --- | --- | --- | --- | --- | --- | --- | --- | --- | --- | --- | --- | --- | --- |
|  | **1** | **2** | **3** | **4** | **5** | |  |  |  |  |  |  |  |  |  |  |  |  |
| **Factor 1 (total score range: 16–80)** | | | | | | 29.2 (9.9) | | 0.8 | | 3.7 | | 37.0 | | 1.0 | | 0.87 | 0.82 | |
| **q14** | 25.4 | 38.8 | 24.8 | 8.7 | 2.3 | 2.2 (1.0) | | 0.5 | | 2.8 | | 25.3 | | 2.3 | | 0.64 | 0.96 | |
| **q15** | 36.5 | 40.8 | 20.0 | 1.7 | 1.0 | 1.8 (0.8) | | 0.7 | | 3.4 | | 36.5 | | 0.9 | | 0.61 | 0.96 | |
| **q16** | 31.0 | 43.8 | 21.5 | 3.1 | 0.6 | 1.9 (0.8) | | 0.5 | | 3.0 | | 30.9 | | 0.5 | | 0.64 | 0.96 | |
| **q17** | 28.1 | 38.7 | 25.8 | 6.3 | 1.2 | 2.1 (0.9) | | 0.5 | | 2.7 | | 28.0 | | 1.1 | | 0.70 | 0.96 | |
| **q18** | 37.9 | 40.6 | 16.7 | 4.0 | 0.8 | 1.8 (0.8) | | 0.8 | | 3.4 | | 37.8 | | 0.7 | | 0.71 | 0.96 | |
| **q19** | 37.5 | 43.5 | 16.5 | 2.3 | 0.2 | 1.8 (0.7) | | 0.6 | | 3.0 | | 37.5 | | 0.1 | | 0.66 | 0.96 | |
| **q20** | 65.4 | 23.7 | 8.7 | 1.3 | 1.0 | 1.4 (0.7) | | 1.8 | | 6.5 | | 65.3 | | 0.9 | | 0.61 | 0.96 | |
| **q21** | 62.1 | 27.9 | 7.9 | 1.7 | 0.4 | 1.5 (0.7) | | 1.5 | | 5.4 | | 62.1 | | 0.3 | | 0.63 | 0.96 | |
| **q22** | 50.2 | 36.9 | 11.2 | 1.2 | 0.6 | 1.6 (0.7) | | 1.1 | | 4.4 | | 50.1 | | 0.5 | | 0.65 | 0.96 | |
| **q23** | 53.7 | 31.3 | 12.9 | 1.5 | 0.6 | 1.6 (0.8) | | 1.1 | | 4.1 | | 53.6 | | 0.5 | | 0.64 | 0.96 | |
| **q24** | 49.0 | 37.3 | 11.9 | 1.5 | 0.2 | 1.6 (0.7) | | 0.9 | | 3.5 | | 49.0 | | 0.1 | | 0.68 | 0.96 | |
| **q26** | 43.7 | 39.4 | 14.8 | 1.9 | 0.2 | 1.7 (0.7) | | 0.7 | | 3.1 | | 43.6 | | 0.1 | | 0.65 | 0.96 | |
| **q27** | 40.8 | 41.0 | 16.0 | 1.7 | 0.6 | 1.8 (0.8) | | 0.8 | | 3.5 | | 40.7 | | 0.5 | | 0.64 | 0.96 | |
| **q28** | 41.0 | 40.6 | 16.2 | 1.9 | 0.4 | 1.8 (0.8) | | 0.7 | | 3.2 | | 40.9 | | 0.3 | | 0.67 | 0.96 | |
| **q29** | 32.5 | 46.9 | 18.5 | 1.2 | 1.0 | 1.9 (0.7) | | 0.7 | | 3.9 | | 32.5 | | 0.9 | | 0.66 | 0.96 | |
| **q30** | 31.9 | 41.2 | 19.8 | 4.4 | 2.7 | 2.0 (0.9) | | 0.9 | | 3.7 | | 31.9 | | 2.6 | | 0.68 | 0.96 | |
| **Factor 2 (total score range: 11–55)** | | | | | | | 28.6 (7.3) | | 0.2 | | 3.2 | | 3.0 | 2.0 | 0.83 | | 0.78 |  |
| **q1** | 6.2 | 25.4 | 49.2 | 16.0 | 3.3 | | 2.8 (0.8) | | 0.0 | | 3.1 | | 6.1 | 3.2 | 0.53 | | 0.96 |  |
| **q2** | 9.0 | 33.5 | 46.7 | 9.0 | 1.7 | | 2.6 (0.8) | | 0.1 | | 3.1 | | 9.0 | 1.7 | 0.62 | | 0.96 |  |
| **q3** | 13.8 | 38.8 | 38.3 | 7.5 | 1.5 | | 2.4 (0.8) | | 0.2 | | 2.9 | | 13.8 | 1.5 | 0.56 | | 0.96 |  |
| **q4** | 15.6 | 30.8 | 32.1 | 16.2 | 5.4 | | 2.6 (1.0) | | 0.2 | | 2.4 | | 15.5 | 5.3 | 0.49 | | 0.96 |  |
| **q6** | 20.4 | 36.7 | 31.0 | 9.0 | 2.9 | | 2.3 (0.9) | | 0.4 | | 2.8 | | 20.3 | 2.8 | 0.58 | | 0.96 |  |
| **q7** | 5.8 | 23.7 | 37.9 | 26.3 | 6.3 | | 3.0 (0.9) | | -0.0 | | 2.5 | | 5.7 | 6.3 | 0.52 | | 0.96 |  |
| **q8** | 27.3 | 38.7 | 25.6 | 7.9 | 0.6 | | 2.1 (0.9) | | 0.4 | | 2.5 | | 27.3 | 0.5 | 0.62 | | 0.96 |  |
| **q9** | 8.3 | 31.9 | 42.7 | 13.1 | 4.0 | | 2.7 (0.9) | | 0.2 | | 2.9 | | 8.2 | 4.0 | 0.58 | | 0.96 |  |
| **q10** | 11.0 | 30.8 | 38.5 | 15.4 | 4.4 | | 2.7 (0.9) | | 0.1 | | 2.6 | | 10.9 | 4.4 | 0.62 | | 0.96 |  |
| **q12** | 14.4 | 30.0 | 37.7 | 12.9 | 5.0 | | 2.6 (1.0) | | 0.2 | | 2.6 | | 14.4 | 5.0 | 0.66 | | 0.96 |  |
| **q13** | 15.4 | 36.0 | 38.5 | 8.5 | 1.7 | | 2.4 (0.9) | | 0.2 | | 2.8 | | 15.3 | 1.7 | 0.67 | | 0.96 |  |
| **Factor 3 (total score range: 6–30)** | | | | | | | 14.3 (4.8) | | 0.5 | | 3.3 | | 24.0 | 4.0 | 0.74 | | 0.81 |  |
| **q45** | 23.1 | 30.4 | 27.1 | 12.5 | 6.9 | | 2.4 (1.1) | | 0.4 | | 2.3 | | 23.0 | 6.9 | 0.50 | | 0.96 |  |
| **q46** | 26.2 | 36.3 | 27.1 | 6.9 | 3.5 | | 2.2 (1.0) | | 0.6 | | 2.9 | | 26.1 | 3.4 | 0.62 | | 0.96 |  |
| **q47** | 30.2 | 37.3 | 25.0 | 4.0 | 3.5 | | 2.1 (1.0) | | 0.7 | | 3.4 | | 30.1 | 3.4 | 0.57 | | 0.96 |  |
| **q48** | 17.5 | 39.2 | 31.5 | 8.7 | 3.1 | | 2.4 (0.9) | | 0.4 | | 2.9 | | 17.5 | 3.0 | 0.63 | | 0.96 |  |
| **q49** | 29.8 | 40.6 | 19.4 | 6.7 | 3.5 | | 2.1 (1.0) | | 0.8 | | 3.3 | | 29.8 | 3.4 | 0.61 | | 0.96 |  |
| **q50** | 12.3 | 26.5 | 32.3 | 18.5 | 10.4 | | 2.8 (1.1) | | 0.1 | | 2.2 | | 12.3 | 10.3 | 0.50 | | 0.96 |  |
| **Factor 4 (total score range: 6–30)** | | | | | | | 12.0 (4.1) | | 0.7 | | 3.7 | | 48.0 | 1.0 | 0.81 | | 0.80 |  |
| **q31** | 23.5 | 44.6 | 26.3 | 4.2 | 1.3 | | 2.1 (0.8) | | 0.5 | | 3.2 | | 23.4 | 1.3 | 0.66 | | 0.96 |  |
| **q32** | 35.0 | 39.6 | 19.2 | 3.8 | 2.3 | | 1.9 (0.9) | | 0.9 | | 3.7 | | 35.0 | 2.3 | 0.58 | | 0.96 |  |
| **q33** | 27.3 | 46.3 | 21.9 | 3.5 | 1.0 | | 2.0 (0.8) | | 0.6 | | 3.4 | | 27.3 | 0.9 | 0.70 | | 0.96 |  |
| **q34** | 35.8 | 42.7 | 19.4 | 1.2 | 1.0 | | 1.8 (0.8) | | 0.7 | | 3.6 | | 35.7 | 0.9 | 0.58 | | 0.96 |  |
| **q35** | 37.1 | 41.0 | 19.2 | 2.3 | 0.4 | | 1.8 (0.8) | | 0.6 | | 2.9 | | 37.1 | 0.3 | 0.66 | | 0.96 |  |
| **q36** | 27.9 | 40.4 | 24.6 | 6.0 | 1.2 | | 2.1 (0.9) | | 0.5 | | 2.9 | | 27.8 | 1.1 | 0.68 | | 0.96 |  |
| **Factor 5 (total score range: 5–25)** | | | | | | | 12.0 (4.3) | | 0.3 | | 2.7 | | 44.0 | 2.0 | 0.81 | | 0.80 |  |
| **q37** | 24.0 | 38.1 | 27.1 | 8.5 | 2.3 | | 2.2 (0.9) | | 0.5 | | 2.8 | | 24.0 | 2.3 | 0.75 | | 0.96 |  |
| **q38** | 21.9 | 34.4 | 31.0 | 10.2 | 2.5 | | 2.3 (1.0) | | 0.3 | | 2.6 | | 21.9 | 2.5 | 0.75 | | 0.96 |  |
| **q39** | 24.0 | 36.7 | 29.0 | 7.9 | 2.3 | | 2.2 (0.9) | | 0.4 | | 2.8 | | 24.0 | 2.3 | 0.73 | | 0.96 |  |
| **q41** | 20.6 | 33.5 | 28.5 | 12.5 | 5.0 | | 2.4 (1.1) | | 0.4 | | 2.5 | | 20.5 | 5.0 | 0.62 | | 0.96 |  |
| **q43** | 19.8 | 25.0 | 32.7 | 15.6 | 6.9 | | 2.6 (1.1) | | 0.2 | | 2.2 | | 19.8 | 6.9 | 0.55 | | 0.96 |  |
